# Experimental verification and validation of immune biomarkers based on chromatin regulators in ischemic stroke

**DOI:** 10.3389/fgene.2022.992847

**Published:** 2022-08-29

**Authors:** Beibei Yu, Yunze Tian, Yongfeng Zhang, Boqiang Lv, Jianzhong Li, Shouping Gong

**Affiliations:** ^1^ Department of Neurourgery, the Second Affiliated Hospital of Xi’an Jiao Tong University, Xi’an, China; ^2^ Department of Thoracic Surgery, the Second Affiliated Hospital of Xi’an Jiao Tong University, Xi’an, China

**Keywords:** ischemic stroke, immune infiltration, chromatin regulators, biomarker, bioinformatics

## Abstract

Ischemic stroke (IS) is a disease characterized by rapid progression and high mortality and disability rates. Its pathophysiological process is inseparable from immune dysfunction. Recently, chromatin regulators (CRs) have been described as a class of enzymes that can recognize, form, and maintain the epigenetic state of an organism, and are closely associated with immune regulation. Nevertheless, the role of CR-related genes in IS has not been fully elucidated. In this study, seven CR-related immune biomarkers in the GSE58294 and GSE22255 datasets were identified by combining differential gene expression analysis, weighted correlation network analysis, and single sample gene set enrichment analysis. After experimental validation using quantitative polymerase chain reaction, four genes (*DPF2, LMNB1, MLLT3*, and *JAK2*) were screened as candidate immune biomarkers. These four biomarkers demonstrated good predictive power in the clinical risk model (area under the curve, 0.775). Molecular docking simulations revealed that mevastatin, WP1066, cladribine, trichostatin A, mequitazine, and zuclomiphene may be potential immunomodulatory drugs for IS. Overall, the results of this study contribute to the identification of CR-related immune therapeutics target in IS and provide an important reference for further research.

## Introduction

Stroke is the second leading cause of death and disability, killing >5.5 million individuals annually (Lindsay et al., 2019). Among stroke types, ischemic stroke (IS) accounts for approximately 71–87% of cases and is caused mainly by blockage of blood flow in the brain (Strong et al., 2007; Campbell et al., 2019). The current treatment for IS focuses on achieving rapid reperfusion through intravenous thrombolysis and/or endovascular thrombectomy. Although these treatments can reduce disability, patients continue to experience serious economic and social burdens (Rochmah et al., 2021). Recent studies have reported that IS can induce immune-inflammatory responses, which in turn aggravate neurological deficits and increase patient mortality (Yu et al., 2020). As such, there is an urgent need to explore the influence of the immune system on IS and to identify potential therapeutic targets.

Chromatin regulators (CRs) are a class of enzymes with special structural functions that can recognize, form, and maintain the epigenetic state of the organism (Lu et al., 2018). Somatic alterations or abnormal expression of CRs may lead to fatal diseases including glioma, lung cancer, bladder cancer, and other tumors. At the same time, CRs are also considered to be important therapeutic targets in diseases such as colitis, and congenital heart disease (Li et al., 2022; Linglart and Bonnet, 2022). In addition, recent research has also found that modulation of these epigenetic genes could improve cellular immune responses in immunotherapy (Belk et al., 2022). Consequently, actively exploring the role of CR-related genes will help to better understand the regulatory role of the immune system in IS.

**FIGURE 1:**
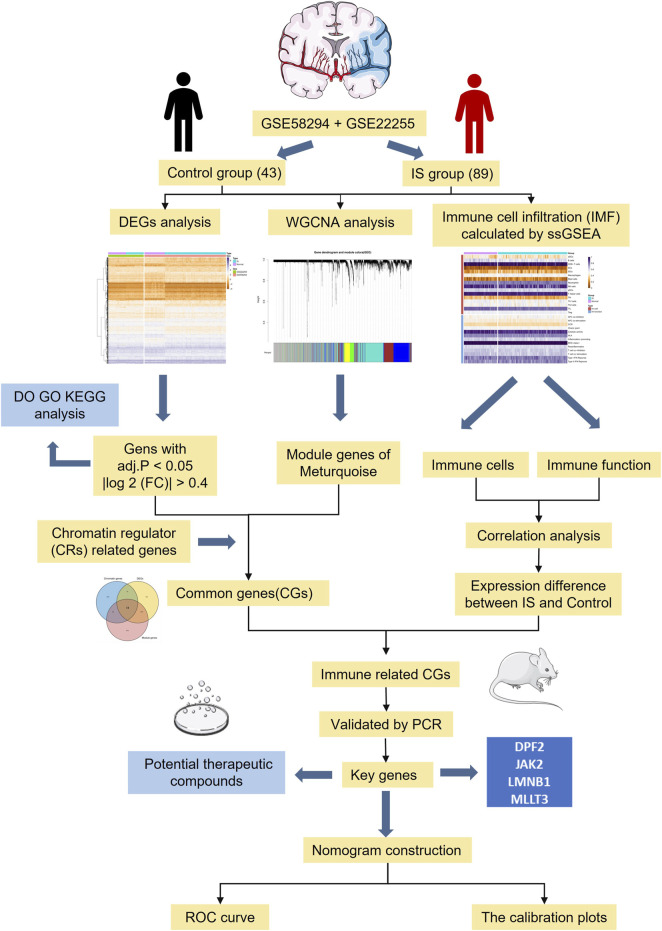
The flowchart of data preparation and analysis.

With the development of high-throughput sequencing and gene chip technologies, bioinformatics can be used to conduct extensive and in-depth analyses of messenger RNA (mRNA) expression profiles of IS(Cao et al., 2021a). In this study, we identified key CR-related immune biomarkers in the GSE58294 and GSE22255 datasets by combining differential gene expression analysis, weighted correlation network analysis (WGCNA), and single-sample gene set enrichment analysis (ssGSEA). After experimental validation of a middle cerebral artery occlusion (MCAO) model, we evaluated the accuracy of key immune therapeutics target in predicting the occurrence of IS. Results of this study may provide a theoretical molecular basis for the diagnosis and targeted treatment of IS. A flow-diagram illustrating our research process is presented in Figure 1.

## Methods

### Establishment of the MCAO model

Twelve specific pathogen-free male Sprague-Dawley rats (weight 280 g) were provided by the Medical Experimental Animal Center (Xi’an Jiaotong University). The Xian Jiaotong University Medical College and Xian Jiaotong University Experimental Animal Center approved all protocols involving animal models or specimens. The rat MCAO model was established based on the modified Zea-Longa model, which removed coil occlusion after *2* h (Longa et al. , 1989). Thereafter, a total of 12 rats were randomly assigned to sham and the IS groups (n = 6 each). Two hours after MCAO, the neurobehavioral scores of the rats in each group were evaluated according to the Longa scale (Longa et al., 1989). Animals that did not exhibit neurological deficits after surgery were excluded. After 3 days of reperfusion, three rats in each group were euthanized using sodium pentobarbital (30 mg/kg) injected through the tail vein. After the brains were removed and sliced, specimens were placed in 2% TTC (Solarbio Life Science, Beijing, China) and incubated at 37°C for 30 min.

### GEO dataset screening

Two publicly available GEO datasets, GSE58294 (Stamova et al., 2014) and GSE22255 (Krug et al., 2012), were used to identify differentially expressed genes (DEGs) between those with IS and healthy individuals (Table 1). GSE58294 contains the whole blood mRNA expression profiles of 69 IS patients and 23 healthy individuals, while GSE22255 contains data from 20 IS patients and 20 healthy individuals.

**TABLE 1:** Detailed information of the gene expression matrixes and platform.

GEO dataset	Platform	Country	Author	Stroke	Normal	Type
GSE58294	GPL570	USA	Stamova et al.	69	23	mRNA
GSE22255	GPL570	Portugal	Krug et al.	20	20	mRNA

### Identification of DEGs and key module genes

Statistical analysis was performed using R version 4.1.0 (R Foundation for Statistical Computing, Vienna, Austria). To analyze microarray data, the gene expression matrix was quantile-normalized and log_2_-transformed after merging it with the probe data (GPL570). Batch effects were removed from the gene expression profiles by merging these two matrices using the comBat algorithm in the sva package of R. The “LIMMA” package was used to identify DEGs, and the filter was |log_2_ (fold-change) > 0.4 and adjusted *p*-value < 0.05.

To explore interactions between genes, the “WGCNA” package was used to perform WGCNA and identify the key module. Thereafter, the “pickSoftThreshold” function was used to obtain the optimal value of the adjacent function weighting parameters, which were used as a soft threshold for subsequent network construction. The related gene modules were then constructed based on hierarchical clustering of the dissimilarity measure. Finally, the correlation between each module and the sample trait (presence or absence of IS) was assessed. Modules with the highest correlation coefficients were selected for further analysis.

### Screening of CR-related biomarkers

A total of 870 CR-related genes was obtained from the supplemental information published by Lu et al. (2018). To identify CRs-related biomarkers, Venn analysis was used to determine the intersection of the above three gene sets, DEGs, the key module genes derived from WGCNA, and CR-related genes. Gene Ontology (GO), Kyoto Encyclopedia of Genes and Genomes (KEGG) pathway, and disease enrichment (DO) analyses were performed on DEGs using the “clusterProfiler” package to identify the underlying pathogenesis and biological pathways of IS.

### Immune infiltration analysis

The degree of immune cell infiltration in each sample was calculated using the ssGSEA algorithm. After downloading the gene set data in “gmt” format with 29 immune-related score, the “GSVA” package was used to score each sample in GSE58294 and GSE22255. Subsequently, the differences in the expression of 16 immune cells and 13 immune-related functions between the IS and healthy samples were further distinguished. Finally, Spearman’s correlation analysis was performed to calculate the correlation coefficients between the CR-related biomarkers obtained in the previous step. Screening criteria for key immune biomarkers related to IS were as follows: > 1/5 of immune cells and function correlation; correlation coefficient, r > 0.3; and *p* < 0.05.

### Construction and validation of IS risk models

Based on the median expression levels of polymerase chain reaction (PCR)-validated immune biomarkers in the samples, they were divided into high and low expression groups. Subsequently, the “rms” package was used to build nomogram model for predicting the incidence of IS. Finally, the “ROCR” package was used to plot a receiver operating characteristic (ROC) curve and a calibration curve to evaluate the effectiveness of the risk model (Cao et al., 2021b).

More importantly, these immune therapeutics target were uploaded to the DSigDB database (http://dsigdb.tan-lab.org/DSigDBv1.0/) to screen small-molecule compounds for IS (Yoo et al., 2015). The screening criteria included *p* < 0.05 and n ≥ 3. The molecular docking software AutoDock Vina was used to simulate the docking between proteins and polymers.

### Quantitative real-time PCR

After 72 h of reperfusion, three rats from each group were euthanized under anesthesia. Tissue samples were collected from the ischemic penumbra of the rats and immediately stored in liquid nitrogen. Total RNA was extracted from each sample using TRIzol reagent (Invitrogen, United States). The extracted RNA was reverse transcribed into complementary DNA using PrimeScriptTM RT Master Mix (TaKaRa, Japan). The primer sequences are listed in Table 2. Relative mRNA expression was calculated using the 2-ΔΔCt method and compared to that of the control group (GAPDH mRNA expression). Statistical comparisons were performed using the student’s *t*-test; differences with *p* < 0.05 were considered to be statistically significant.

**TABLE 2:** Specific primers used for quantitative real-time PCR.

Primer	Sequence
GAPDH-F	GGT​CGG​TGT​GAA​CGG​ATT​T
GAPDH-R	TGA​ACT​TGC​CGT​GGG​TAG​A
JAK2-F	ACA​CCT​CTG​ATC​CCT​CAG​C
JAK2-R	GCG​AAT​GAT​AAA​CAG​GCA​GGA​TG
MLLT3-F	ACA​ACG​AGG​AGG​AGT​CTG​ATG​AGG
MLLT3-R	CAC​TGT​CAC​TGC​CGT​CAC​TCA​AG
LMNB1-F	AGC​TCT​CTC​CAA​GTC​CTT​CTT​CCC
LMNB1-R	CAC​TAC​TGC​TCG​CCT​CTG​ATT​CTT​C
GLYATL1-F	GCA​GTG​AGA​GGA​GCC​AAC​GAT​TC
GLYATL1-R	ATC​AGA​GCC​CAG​GAC​ACA​GGA​G
DPF2-F	TAA​GCC​AGA​CAC​GGA​CCA​GAC​TC
DPF2-R	CAG​TAC​GCA​GCA​GAG​CCT​CTA​AAC
BRCA1-F	CAG​ATC​GAG​AGT​TGT​GGT​AGC​AGT​G
BRCA1-R	TTG​GCT​CGT​TCT​TCT​TGG​CAT​CAG

## 3 Results

### Identification of DEGs using the LIMMA package

**FIGURE 2:**
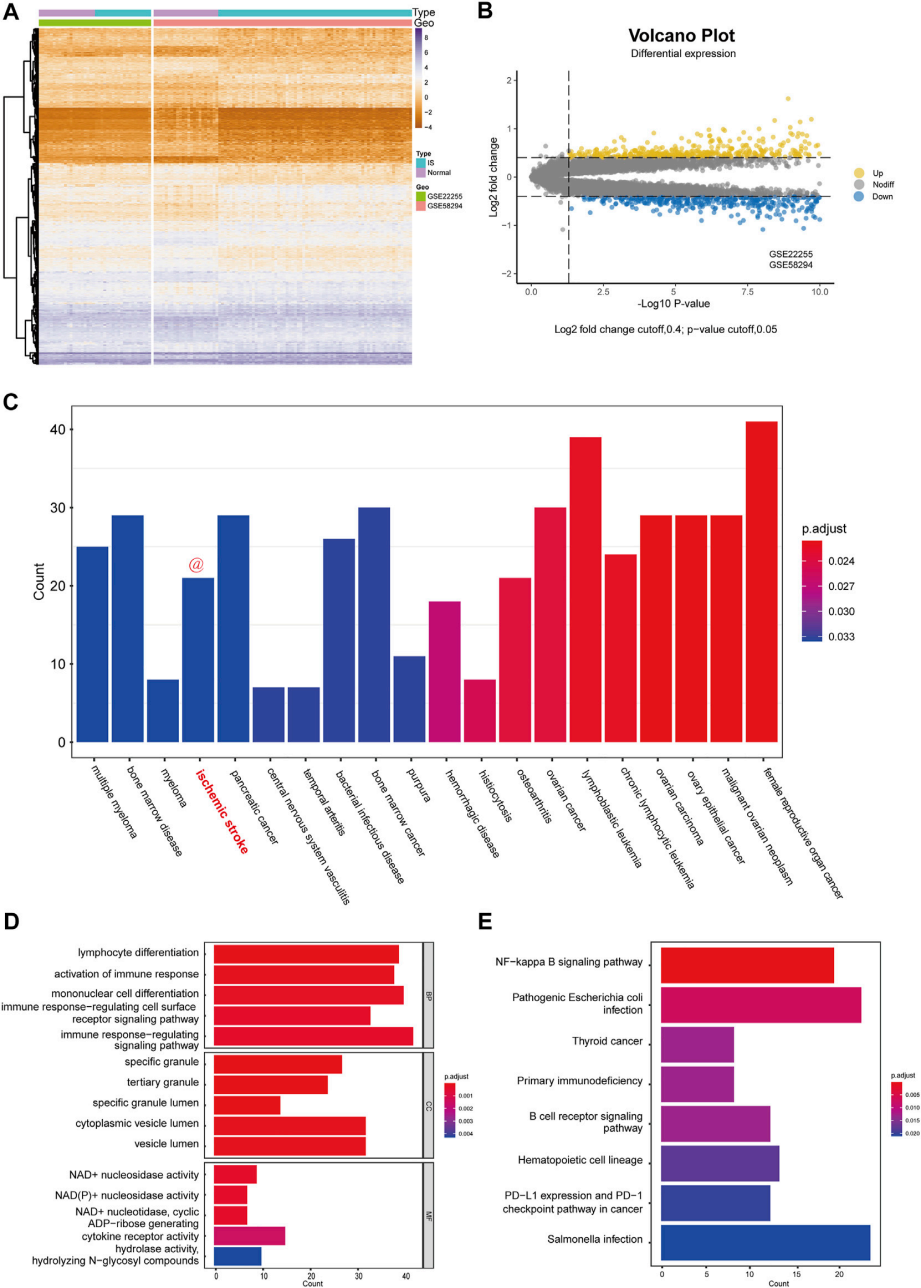
Differential expression analysis and functional enrichment analysis. **(A)** Cluster heatmap for DEmRNAs in GSE58294 and GSE22255 dataset. Yellow represents high gene expression and purple represents low expression. **(B)** Volcano plot for DEmRNAs in GSE58294 and GSE22255 dataset. **(C)** Disease enrichment analysis of DEGs. **(D)** GO functional enrichment analysis of DEGs. **(E)** KEGG pathway analysis of DEGs.

After the successful merging of GSE58294 and GSE22255, 987 genes were differentially expressed, of which 445 were upregulated and 542 were downregulated. The DEGs results are presented in the form of a heatmap and volcano plot (Figure 2A,B). DO analysis revealed that the DEGs were specific to the occurrence of IS (Figure 2C).

To better assess the function of the DEGs, GO functional enrichment (Figure 2D) and KEGG pathway analyses (Figure 2E) were performed. Biological processes (BP) included “lymphocyte differentiation,” “activation of immune response,” “mononuclear cell differentiation,” “immune response-regulating cell surface receptor signaling pathway,” “immune response-regulating signaling pathway,” which reflected that DEGs has a strong correlation with immune function. In addition, cellular components (CC) indicated that DEGs were involved in the release of specific granules of immune cells, such as neutrophils, while molecular functions (MF) indicated that DEGs were involved in the metabolism of NAD^+^/NADH. In addition, KEGG pathway analysis indicated that the DEGs were mostly associated with three pathways: the NF-kappa B signaling pathway; primary immunodeficiency; and B cell receptor signaling pathway.

### Identification of turquoise genes and CRs-related biomarkers

**FIGURE 3:**
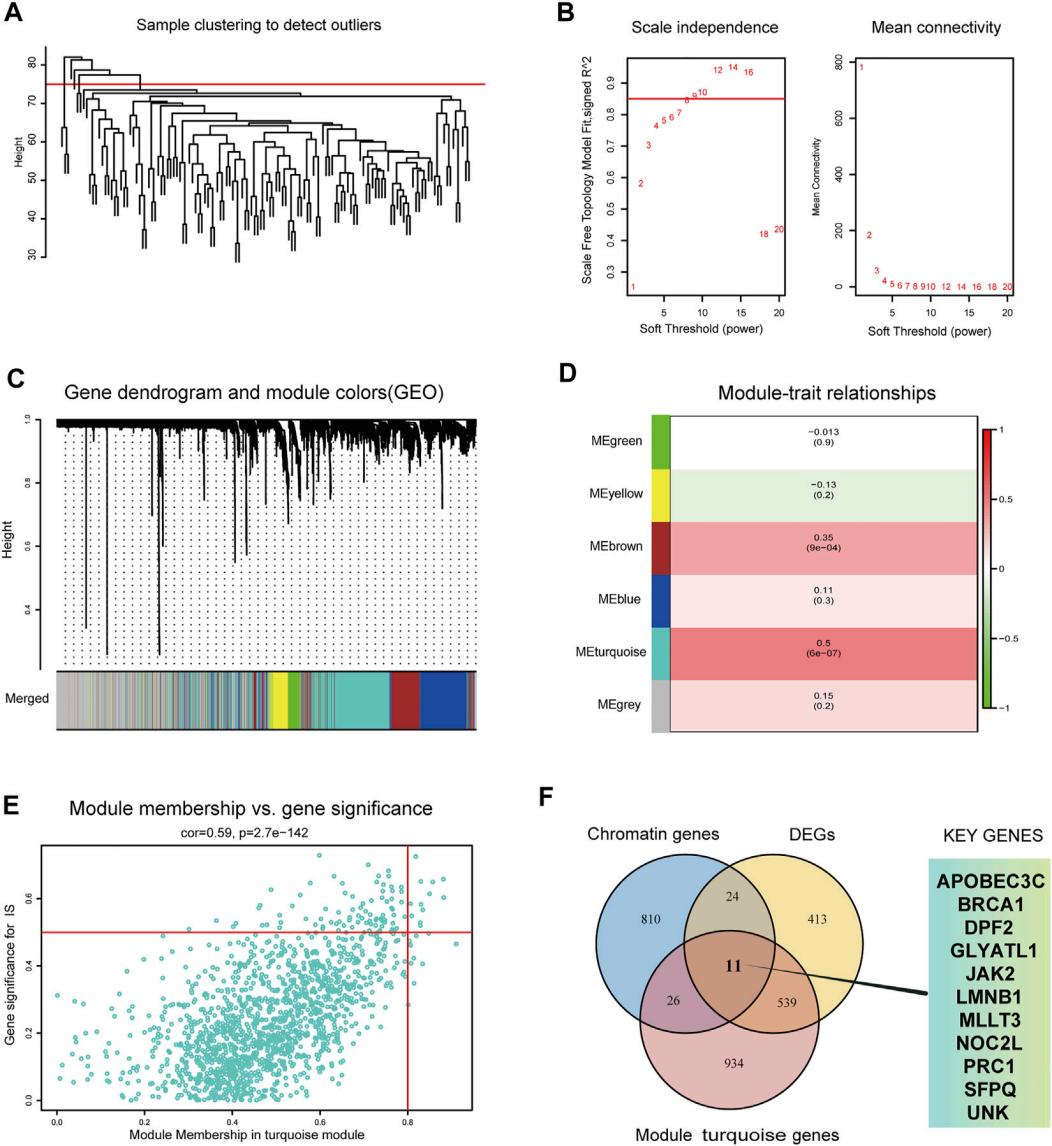
Identification of key modules genes and CRs related biomarkers **(A)** Outlier removal with “h > 75”. **(B)** Soft threshold setting according to R2 = 0.85. **(C)** The gene set is divided into 6 different modules. **(D)** The correlation of modules with IS occurrence. **(E)** The correlation between turquoise module and IS gene significance. **(F)** The Venn plot of the three gene sets, DEGs, turquoise module genes and CRs-related genes.

First, WGCNA was used to select genes with a variance >25%, followed by sample cluster analysis to eliminate outlier samples with h > 75 (Figure 3A). The optimal soft threshold β = 9 was determined according to a scale-free fitting index (R2) of 0.85 (Figure 3B). As shown in Figures 3C,D, WGCNA identified six modules, of which the turquoise module was strongly correlated with IS (module feature correlation = 0.50). Moreover, correlation analysis revealed that the correlation between module membership in turquoise modules and gene significance for IS was 0.59, which was statistically significant (Figure 3E). The intersection of the three gene sets of DEGs, turquoise module genes, and CR-related genes is shown in Figure 3F. A total of 11 overlapping biomarkers related to CRs were identified for further analysis.

### Immune infiltration analysis and immune biomarker screening

**FIGURE 4:**
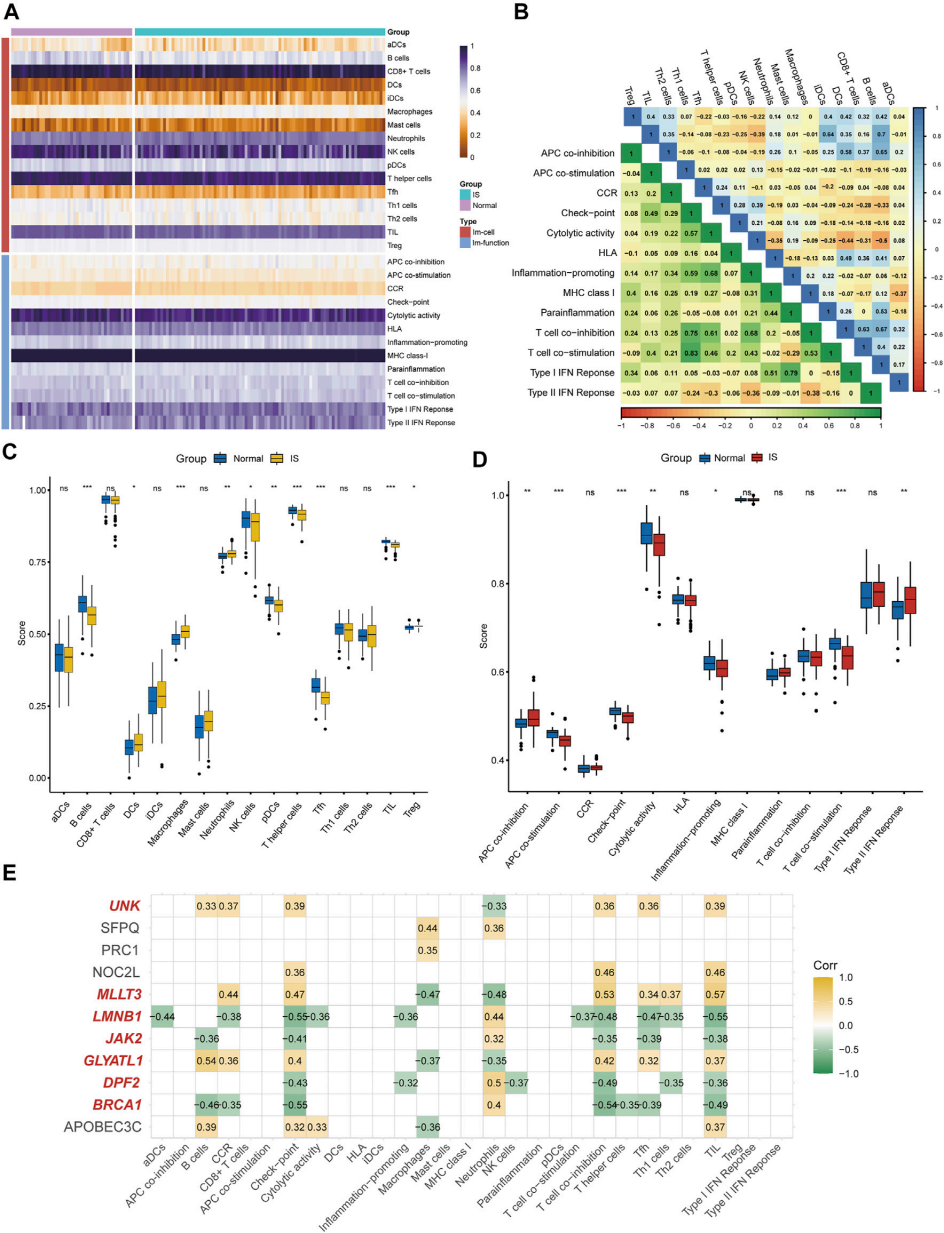
Immune infiltration analysis and immune biomarkers screening. **(A)** Immune infiltration heatmap in GSE58294 and GSE22255 dataset. **(B)** Correlation heatmap of immune cells and immune function. **(C)** The expression difference of 16 immune cells between IS and healthy samples. **(D)** the expression difference of 13 immune-related functions between IS and healthy samples **(E)** Screening for CRs-related immune biomarkers.

According to the ssGSEA algorithm, the immune infiltration scores of 16 immune cells and 13 immune-related functions in 132 samples were evaluated (Figure 4A). For immune cells, B cells had a strong positive correlation (r = 0.64) with follicular helper T cells, whereas neutrophils had a strong negative correlation (r = −0.44) with T-helper one cells (Figure 4B). For immune function, type-I interferon (IFN) response had a strong positive correlation (r = 0.79) with para-inflammation, and type-II IFN response had a strong negative correlation (r = −0.38) with T cell co-inhibition.

Differences in immune cell content and immune function between IS and healthy individuals were further explored (Figures 4C,D). It was clearly shown that the proportion of dendritic cells, macrophages, neutrophils, and regulatory T cells was higher in the IS group than in the control group, and the functions of antigen presenting cell (APC) co-inhibition and type-II IFN response were better than those of healthy individuals. Meanwhile, the proportion of B cells, NK cells, plasmacytoid DC cells, T-helper cells, follicular helper T cells, and tumor-infiltrating lymphocytes in the control group were higher than those in the IS group, and the functions of APC co-stimulation, checkpoint, cytolytic activity, inflammation-promoting, and T cell co-stimulation were greater than those of IS patients. Finally, seven of 11 DEGs (*BRCA1*, *DPF2*, *GLYATL1*, *LMNB1*, *MLLT3*, *JAK2*, and *UNK*) were identified as immune biomarkers based on correlation analysis (Figure 4E).

### Validation of key immune biomarkers

**FIGURE 5:**
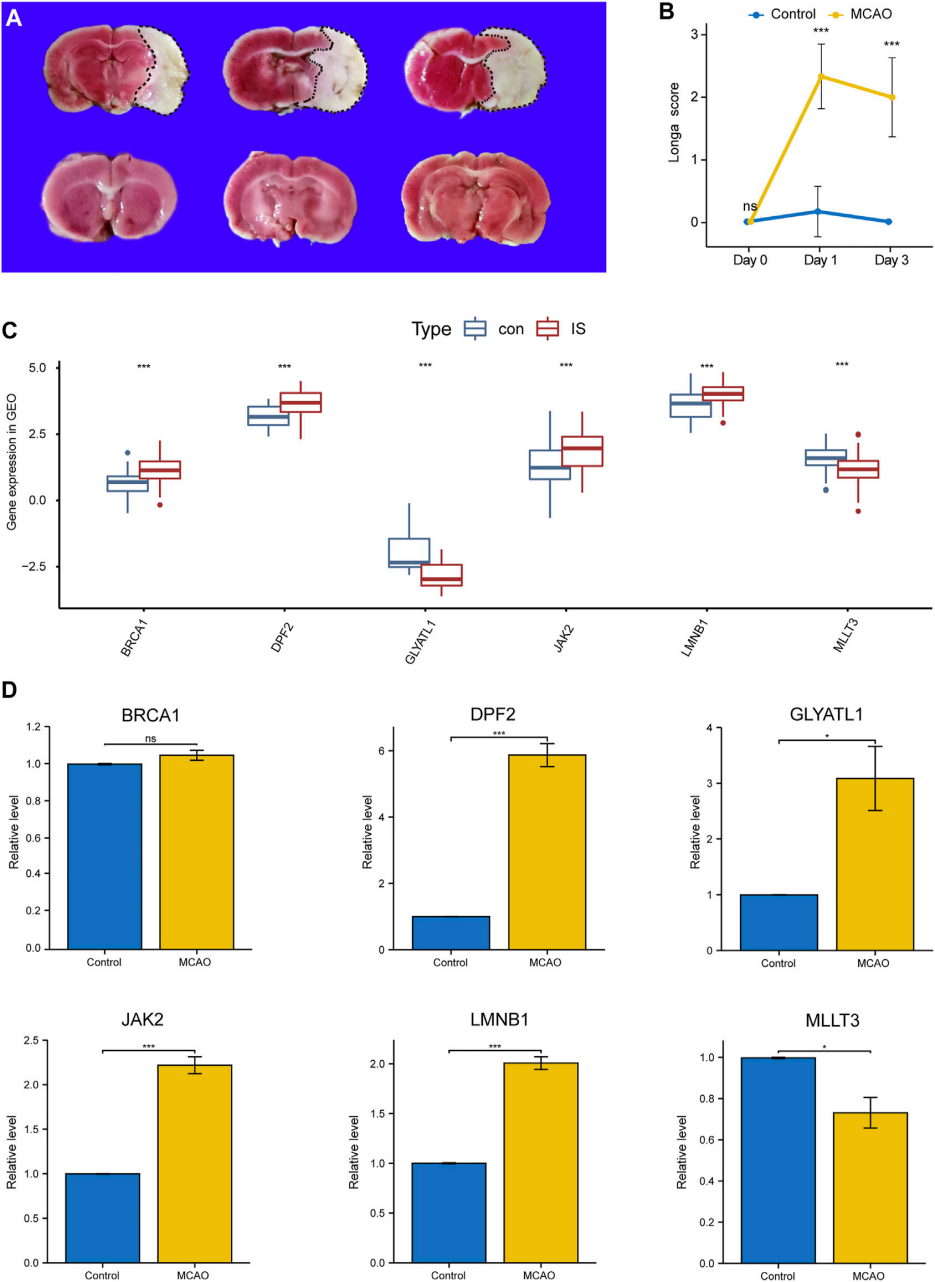
Validation of Key Immune biomarkers by MCAO models. **(A)** TTC staining in Sham group and IS group. **(B)** Longa scores in Sham group and IS group. **(C)** Expression of CRs-related immune biomarkers in GSE58294 and GSE22255 datasets. **(D)** qPCR verifies the expression of CRs-related immune biomarkers.

As shown in Figures 5A,B, compared with the sham group, the IS group exhibted obvious cerebral infarction lesions and increased Longa scores, indicating that the MCAO model was successful. During PCR validation of the seven immune biomarkers, *UNK* was excluded due to its poor species conservation between humans and rats. In the GEO expression matrix, the mRNA expression levels of *BRCA1*, *DPF2*, *LMNB1*, and *JAK2* in the IS group were higher than those in the control group, and *MLLT3* and *GLYATL1* were lower than those in the control group (Figure 5C). Real-time-qPCR (Figure 5D) further demonstrated that the mRNA expression of *DPF2*, *LMNB1*, *MLLT3*, and *JAK2* was consistent with the prediction of bioinformatics analysis and was statistically significant (*p* < 0.05).

### Construction of clinical risk models

**FIGURE 6:**
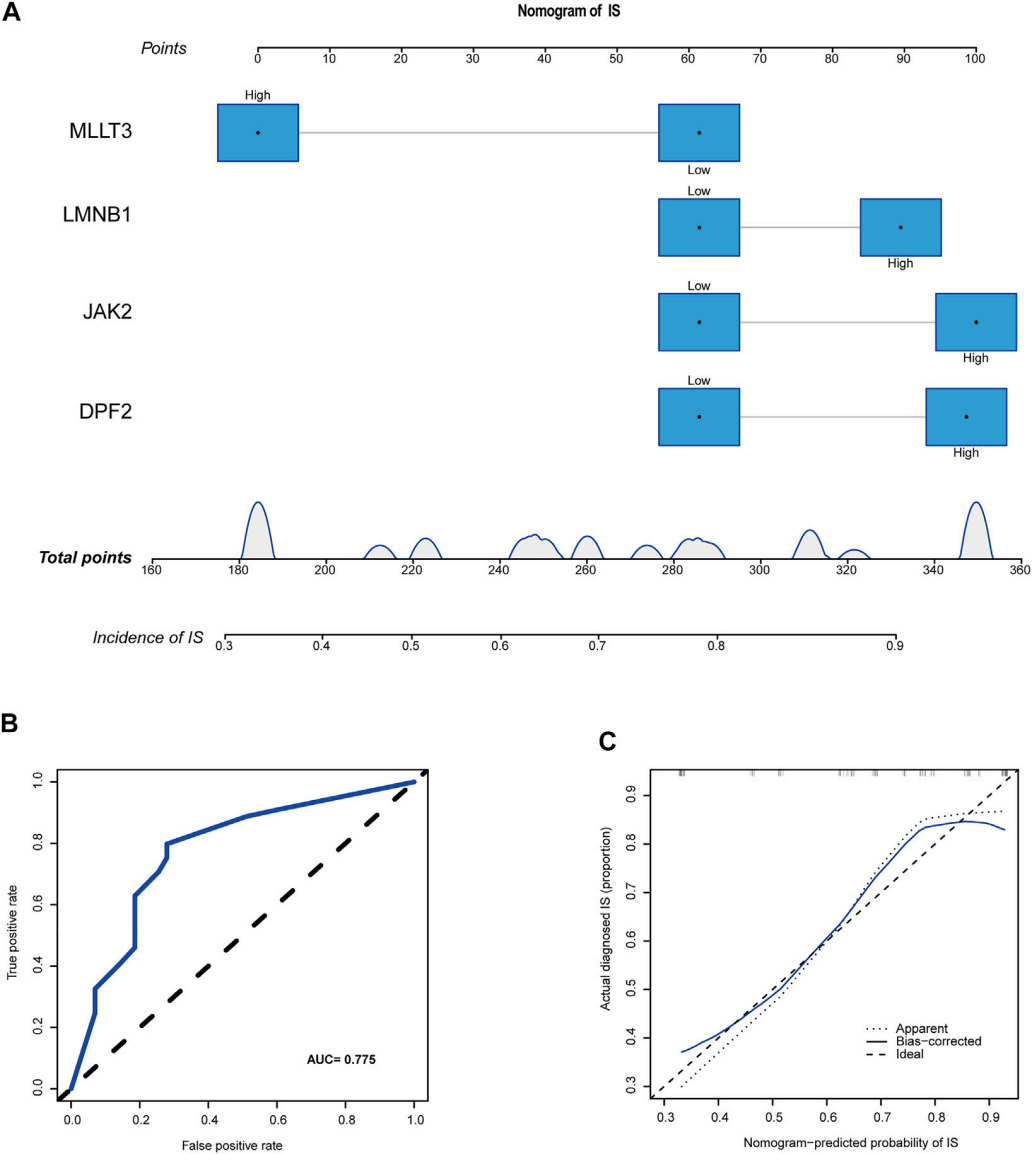
A novel nomogram for predicting IS. **(A)** CRs-related immune biomarkers predict the occurrence of IS. **(B)** ROC curve for nomogram. **(C)** The calibration curve for nomogram.

The median expression levels of *DPF2*, *LMNB1*, *MLLT3*, and *JAK2* were 3.58, 3.93, 1.27, and 1.74, respectively, which were the baseline levels in the multivariate logistic regression analysis. Subsequently, the “rms” package was used to construct a nomogram model for predicting the risk for IS, as shown in Figure 6A. A ROC curve was used to evaluate the internal validation results of the dataset, and its AUC was 0.775 (Figure 6B). Furthermore, good agreement between the estimated values and actual observations was found with the calibration curve.

### Potential therapeutic compounds for IS

**FIGURE 7:**
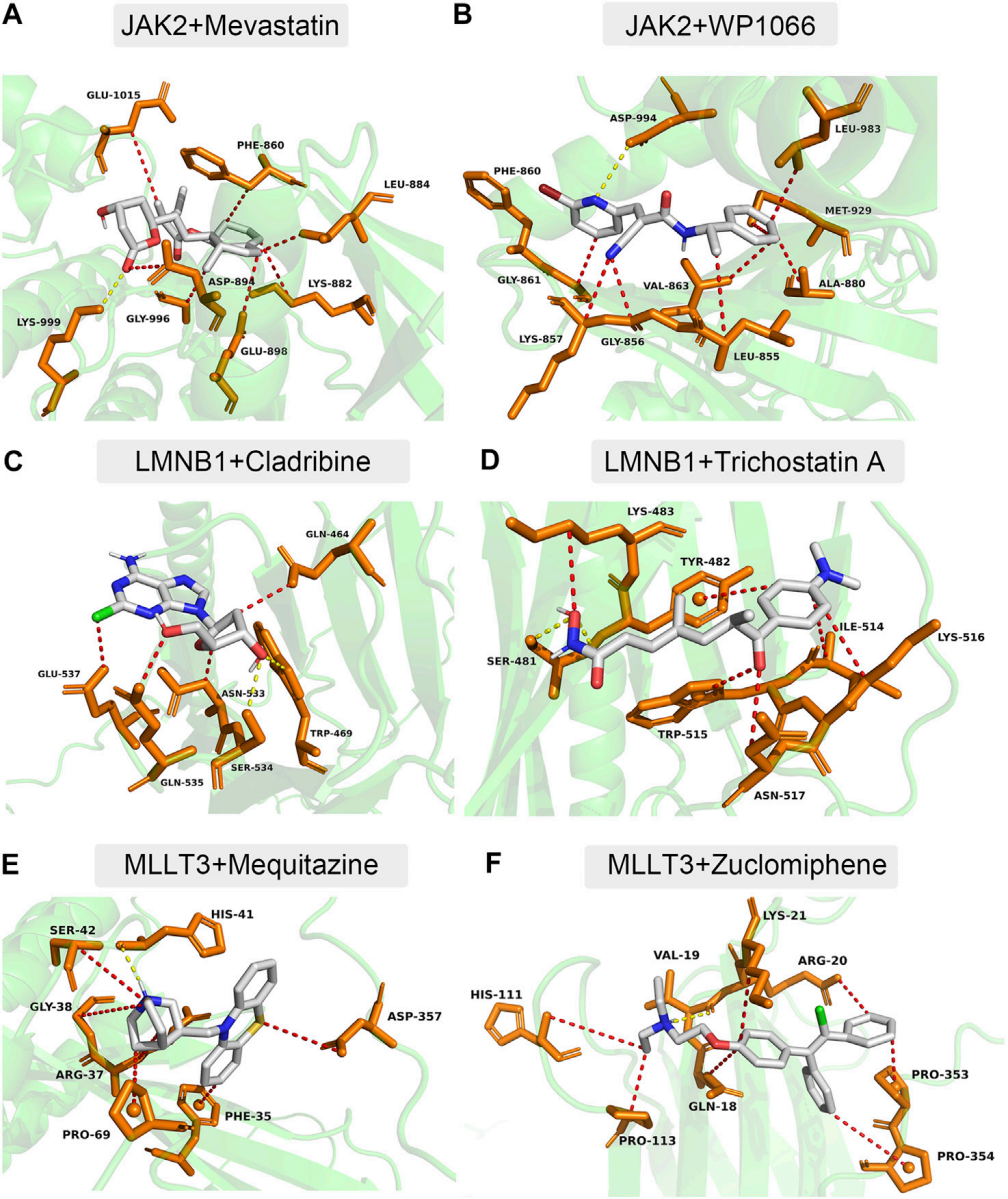
Docking simulation of proteins and small molecule compounds. **(A)** *JAK2* and Mevastatin. **(B)** *JAK2* and WP1066. **(C)** *LMNB1* and Cladribine. **(D)** *LMNB1* and Trichostatin **(A)**. **(E)** *MLLT3* and Mequitazine. **(F)** *MLLT3* and Zuclomiphene.

Using the DSigDB database, 34 small-molecule compounds that may bind to *LMNB1*, *MLLT3*, and *JAK2* were identified. Accordingly, the protein structures of *LMNB1*, *MLLT3*, and *JAK2* in the PDB database were investigated. Following the calculation of binding energies, the top 10% of the small-molecule compounds with the lowest binding energies were retained. Among them, *JAK2* may be combined with mevastatin and WP1066 (Figure 7A,B), *LMNB1* may be combined with cladribine and trichostatin A (Figure 7C,D), and *MLLT3* may be combined with mequitazine and zuclomiphene (Figure 7E,F).

## Discussion

In the complex pathogenesis of IS, lymphoid organs are activated, followed by stroke-induced immunosuppression to reduce inflammatory damage (Lindsay et al., 2019; Westendorp et al., 2022). From an epigenetic perspective, CR-related biomarkers could help shed light on the mechanisms of central nervous system and immune system dysregulation in IS. In this study, we explored four CR-related immune biomarkers associated with IS. To our knowledge, this was the first study to use WGCNA to screen and identify key immune therapeutics target based on CRs in patients who experienced IS. We verified our conclusions using PCR with the MCAO model. Our study provides a rationale and promising research recommendations for the possible epigenetic mechanism(s), treatment, and prognosis of this lethal disease.

Epidemiological data suggest that post-stroke infection is the leading cause of death and disability among patients, and is associated with higher rates of recurrence and readmission (Zhang et al., 2021). The down-regulation of immune system function after IS reduces inflammation on the one hand, but on the other, conversely increases the risk for infection (Qin et al., 2020). Animal experiments have also shown that immune cell therapy can produce significant beneficial effects by improving infarct size and neurological scores in animal models. Consistently, the GO and KEGG results of DEGs in our study indicated that IS was closely associated with the activation of immune response, immune response-regulating signaling pathway, and primary immunodeficiency.

Given the close association between IS and the immune system, we further dissected immune infiltration of the disease using ssGSEA. According to analysis of the immune cell infiltration landscape, the first step of an inflammatory response following IS is the activation of resident microglia, followed by the infiltration of peripheral immune cells such as neutrophils and macrophages. These variations promote neuroinflammation and tissue repair after ischemia. Nathan (2006)showed that brain damage caused by IS was induced by neutrophil-mediated oxidative stress and the release of proteolytic enzymes. In addition, our findings also revealed immunosuppression after IS, which is embodied in the downregulation of B cells, NK cells, plasmacytoid DC cells, T-helper cells, follicular helper T cells, and tumor-infiltrating lymphocytes. Extensive literature suggests that immunosuppression is associated with the post-stroke-induced activation of the sympathetic and parasympathetic nervous systems (Haspula and Clark, 2018; Zera and Buckwalter, 2020).

After validation by qPCR, we screened four biomarkers (*DPF2*, *LMNB1*, *MLLT3*, and *JAK2*) that were most relevant to immunity. These four biomarkers demonstrated good predictive power in the clinical risk model (AUC, 0.775), and which may bring assistance for IS patients with negative MRI imaging. Using a rat model of cerebral ischemia, Wang et al. reported that the *Jak2* inhibitor AG490 could improve neurological deficits, cerebral infarction, edema, oxidative stress, and inflammation (Wang et al., 2021). A recent study also identified *Jak2* as an immune-related gene involved in IS pathophysiology (Wang et al., 2022). However, the roles of *DPF2*, *LMNB1*, and *MLLT3* in IS remain to be explored.

Another vital finding from our study was that several potential therapeutic compounds for the treatment of IS were identified. *JAK2*, *LMNB1*, *MLLT3* and can bind to mevastatin, WP1066, cladribine, trichostatin A, mequitazine, and zuclomiphene. Clinical practice and animal experiments further validated our predictions of immunomodulatory drugs. Some studies have reported that mevastatin, a HMG-CoA reductase inhibitor, can reduce infarction damage (Amin-Hanjani et al., 2001). WP1066 also demonstrated potential to ameliorate ischemic brain injury in rats (Yu et al., 2020). A study by Lingling et al. reported that trichostatin A exerts neuroprotective effects by improving autophagy/lysosomal dysfunction in neurons (Lingling et al., 2022).

It is important to note, however, that the present study had some inherent limitations. First, it was based on bioinformatics analysis and animal experiments, and the results still need to be validated in rat whole blood and clinical IS patients. Second, the sample size of IS patients included in the study was limited. Although the risk model constructed in this study demonstrated good performance, it remains necessary to integrate more sample data to improve the stability of the model. This study also suffered from the inherent drawback of confounding bias in time and space, which include race, region, and time period of IS patients. Finally, the development of immunomodulatory drugs represents a feasible treatment method for IS; however, this needs to be verified in animal experiments, which in turn can be applied to clinical settings.

Collectively, we identified four candidate genes as potential therapeutics target for IS according to bioinformatics analysis and qPCR, and further explored immunomodulatory drugs that may bind to these immune genes related to CRs. Results of the present study contribute to the discovery of CR-related immune therapeutics target in IS and provide an important reference for further research.

## Data Availability

The original contributions presented in the study are included in the article/Supplementary Material, further inquiries can be directed to the corresponding authors.
